# Branch Pulmonary Artery Regurgitation in Repaired Tetralogy of Fallot: Correlation with Pulmonary Artery Morphology, Distensibility, and Right Ventricular Function

**DOI:** 10.3390/tomography7030036

**Published:** 2021-09-01

**Authors:** Suvipaporn Siripornpitak, Duangkanok Lueangwattanapong, Apichaya Sriprachyakul, Suthep Wanitkun, Alisa Limsuwan

**Affiliations:** 1 Department of Diagnostic and Therapeutic Radiology, Faculty of Medicine, Ramathibodi Hospital, Mahidol University, 270 Rama 6 Road, Bangkok 10400, Thailand; duangkanok403@gmail.com (D.L.); apichaya.srp@mahidol.ac.th (A.S.); 2 Division of Cardiology, Department of Pediatrics, Faculty of Medicine, Ramathibodi Hospital, Mahidol University, 270 Rama 6 Road, Bangkok 10400, Thailand; suthep.wan@mahidol.ac.th (S.W.); alisa.lim@mahidol.ac.th (A.L.)

**Keywords:** repaired tetralogy of Fallot, branch pulmonary artery, regurgitation fraction, pulmonary distensibilty, right ventricular function, magnetic resonance imaging

## Abstract

Background: The aim was to determine the effect of pulmonary artery (PA) morphology on the branch pulmonary artery-regurgitation fraction (BPA-RF), the relationship of pulmonary insufficiency (PI) to BPA-RF and PA-distensibility, and factors (BPA-RF and PA-distensibility) associated with right ventricular function (RVF) in repaired tetralogy of Fallot (rTOF). Methods: A total of 182 rTOF patients (median age 17.1 years) were analyzed for length, angle of PA, BPA-RF, PI, and PA-distensibility, using magnetic resonance imaging. Results: The left PA had a significant greater RF than the right PA (median (interquartile range)): LPA 43.1% (32.6–51.5) and RPA 35.2% (24.7–44.7), *p* < 0.001. The LPA was shorter with a narrower angle than the RPA (*p* < 0.001). The anatomy of the branch-PA was not a factor for the greater LPA-RF (odds ratio, 95% confidence interval: CI, *p*-value): length 0.44 (0.95–2.00), *p* = 0.28; angle 0.63 (0.13–2.99), *p* = 0.56. There was a strong positive correlation between PI and BPA-RF-coefficients (95% CI), *p*-value: LPA 0.78% (0.70–0.86), *p* < 0.001; RPA 0.78% (0.71–0.84), *p* < 0.001 and between BPA-RF and distensibility-coefficients (95%CI), *p*-value: LPA 0.73% (0.37–1.09), *p* < 0.001; RPA 1.63% (1.22–2.03), *p* < 0.001, respectively. The adjusted BPA-RF did not predict RVF, RPA (*p* = 0.434), LPA (*p* = 0.268). Conclusions: PA morphology is not a significant factor for the differential BPA-RF. The vascular wall in rTOF patients responds to chronic increased intravascular volume by increasing distensibility. BPA-RF is not a determinant of RVF.

## 1. Introduction

Tetralogy of Fallot is one of the most common cyanotic heart diseases, and requires surgical repair during infancy or early childhood [1]. Definitive repair consists of ventricular septal defect closure and right ventricular outflow augmentation to relieve obstruction [2]. Post-repair sequelae, including pulmonary insufficiency, right ventricular dilatation, biventricular dysfunction, and exercise intolerance, are common in patients with repaired tetralogy of Fallot (rTOF) [3,4]. Pulmonary insufficiency may cause flow changes in both left and right pulmonary arteries (LPA, RPA) and regurgitation in the branch pulmonary arteries. The greater the branch pulmonary artery regurgitation fraction (BPA-RF), the lesser the net forward flow to the lungs. Phase-contrast magnetic resonance imaging (phase-contrast MRI) has been used to evaluate pulmonary valve function and branch pulmonary artery distensibility in healthy subjects [5]. Magnetic resonance imaging (MRI) is also a gold standard to evaluate pulmonary insufficiency and flow dynamics in patients with rTOF [6].

Few studies have been conducted to investigate the causes and consequences of the difference with BPA-RF [7,8,9,10,11]. That BPA-RF is greater in the LPA has been established, with a ratio of regurgitation of LPA: RPA of 40–46%: 29–33% [9,10,11]. However, the cause of differential BPA-RF has not been well elucidated. Harris et al. correlated BPA-RF with pulmonary vascular resistance of both lungs [7]. Voser et al. found that flow dynamics in the branch pulmonary artery were different. The RPA is laminar flow, while the LPA is mainly vortex flow [11]. Pulmonary artery distensibility determines vascular stiffness and impedance. In addition, pulmonary distensibility in patients with rTOF is greater than that found in the normal population, and LPA is stiffer than RPA [11].

The effect of LPA angulation on pulmonary perfusion [12,13] and the clinical implications for interventional angioplasty have been reported [14].

To date, there are few studies that have investigated the cause of the differential BPA-RF and its relationship with pulmonary insufficiency and branch pulmonary distensibility. The effects of BPR-RF and branch pulmonary distensibility on right ventricular function have not been fully established. The purpose of this study is to assess the effect of pulmonary artery morphology on the differential BPA-RF, to test the relationship between pulmonary insufficiency and BPA-RF and vascular distensibility, and to determine factors associated with right ventricular function, using MRI.

## 2. Materials and Methods

### 2.1. Study Population and Data Collection

This retrospective study included 294 patients with definitive surgical correction for a preoperative diagnosis of tetralogy of Fallot or double outlet right ventricle of tetralogy of Fallot subtype, who underwent MRI examination at the radiology department in our institution from January 2009 to July 2019. One hundred and twelve patients were excluded for the following reasons: rTOF with percutaneous or surgical pulmonary valve replacement (n = 22), rTOF with Rastelli operation (n = 2), significant pulmonary artery stenosis which was defined as a greater than 50% narrowing diameter compared with the adjacent vessel proximally (n = 63) [15], moderate to severe pulmonary valve stenosis which was defined as a peak velocity across the valve greater than 3 m/s (n = 1) [16], and incomplete MRI data (n = 24). Thus, a total of 182 patients were included in the analysis.

Patient demographic information, age, weight, height, body surface area at the time of the MRI study, age at definitive repair, and mode of surgery were collected from the medical records. Hemodynamic data, right ventricular ejection fraction, pulmonary insufficiency, and BPA-RF were obtained from the MRI report.

### 2.2. MRI Protocol

MRI studies were performed using one of two models of a 3.0-tesla scanner (Philips, Best, The Netherlands). Between 2009 and 2013, the Intera Achieva model was used, and from 2014 to 2019, the Ingenia model. Images of RPA and LPA were obtained from contrast-enhanced magnetic resonance angiography. The image parameters were as follows: echo time/repetition time/flip angle of 1.63 ms/4.9 ms/30 degrees, bandwidth of 1, field of view of 340 mm, matrix of 380 × 226, slice thickness/reconstruction thickness of 3 mm/1.5 mm. Data were collected on the coronal plane using CENTRA k-space data collection. Gadolinium contrast agent (Magnevist or Gadovist, Bayer Schering, Leverkusen, Germany) 0.1 mmol/kg (Gadovist) to 0.2 mmol/kg (Magnevist) was injected intravenously. A real-time bolus track technique was used to determine the appropriate time for image acquisition.

A through-plane phase-contrast MRI was used to calculate the regurgitation fraction. The image parameters were as follows: a slice thickness of 6–8 mm, echo time/repetition time/flip angle of 2.3 ms/2.8 ms/10 degrees, a spatial resolution of 2.0 × 2.0 to 2.2 × 2.2 mm, a matrix of 128 × 128 to 132 × 124, velocity encoding of 180–200 cm/s at main pulmonary artery and 150–180 cm/s at both RPA and LPA, and 30–40 cardiac phases throughout the cardiac cycle. The level of the prescribed image was as follows: main pulmonary artery was at its midpoint between the pulmonary valve and pulmonary artery bifurcation, RPA was at approximately 2/3 distal to its origin, and LPA was at about the midpoint from its origin. Data were acquired after breath-hold, at expiration.

MRI examination on children under 11 years of age was performed under general anesthesia by an anesthesiologist.

### 2.3. Image Analysis

**Figure 1:**
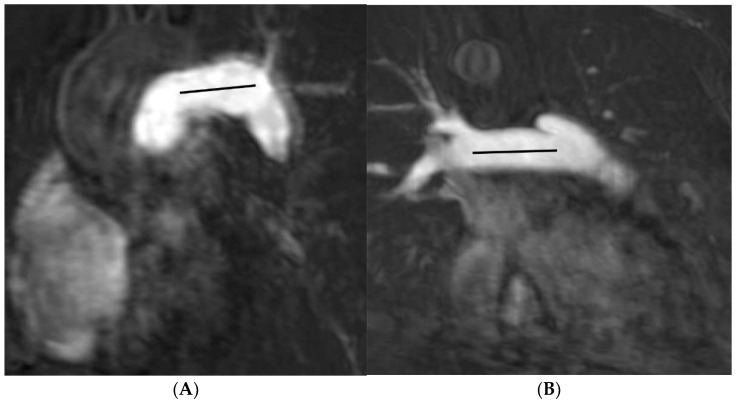
Contrast-enhanced magnetic resonance angiography in the left and right anterior oblique planes demonstrates measurement of the length of left pulmonary artery (**A**) and right pulmonary artery (**B**).

**Figure 2:**
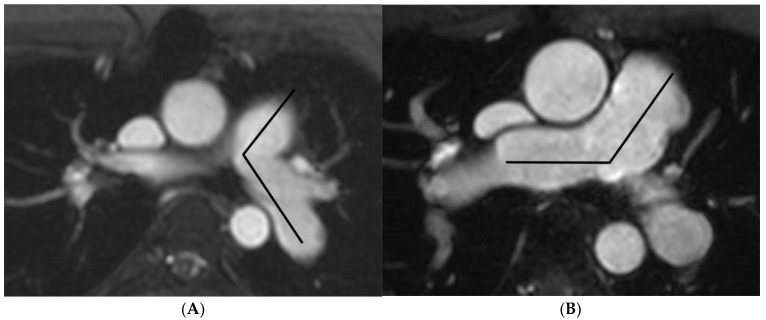
Contrast-enhanced magnetic resonance angiography in axial plane demonstrates measurement of the angle of left pulmonary artery (**A**) and right pulmonary artery (**B**).

**Figure 3:**
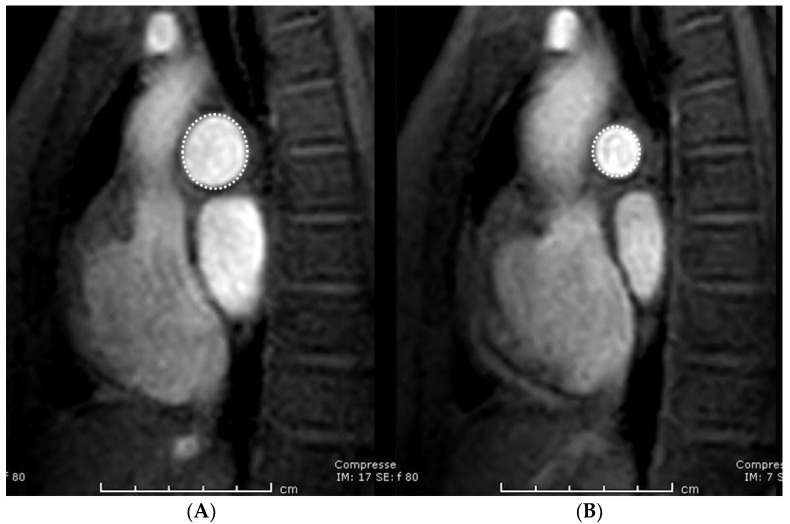
Phase-contrast magnetic resonance imaging of the pulmonary artery demonstrates the measurement of maximum area (**A**) and minimum area (**B**).

MRI images were uploaded into the Picture Archiving and Communications System (PACS), using DICOM Conformance (Synapse PACS version 3.2.0, FUJIFILM Medical Systems U.S.A., Inc, Stamford, CT, USA). The contrast-enhanced magnetic resonance angiography images in the coronal plane were reconstructed into axial, and right and left anterior oblique planes following the long-axis of RPA and LPA, respectively. Morphology of the pulmonary artery (length and angulation) was obtained from the contrast-enhanced magnetic resonance angiography images. The length of RPA and LPA was defined as the distance between the origin and the lobar branch of the upper lobe of each artery [17]. Images from the four reconstruction planes were used to determine the origins of the pulmonary artery and the lobar artery. The measurements were obtained from the left and right anterior oblique planes, respectively (Figure 1A,B). The angle measurement was obtained from the axial plane. Pulmonary artery angulation was defined by measuring the angle between the midline of the LPA or RPA and the midline of main pulmonary artery [14]. Measurement was shown in (Figure 2A,B). Measurement of the maximum and minimum areas of both RPA and LPA was obtained from phase-contrast MRI images (Figure 3A,B). The percentage of pulmonary distensibility was defined as (maximum area-minimum area) × 100/minimum area of each pulmonary artery [5].

Commercial software (ViewForum Release 4.1 and Extended MR Workspace 2.6.3.5; Philips, Best, The Netherlands) was used for post-processing and calculation of pulmonary regurgitation. The pulmonary artery area was traced from the magnitude image by using semi-automated contouring software. Pulmonary regurgitation was calculated by direct measurement of the retrograde flow compared with the antegrade flow and expressed as the regurgitation fraction (%). The severity of branch pulmonary regurgitation and pulmonary insufficiency was evaluated by using regurgitation fraction values. The regurgitation fraction was classified into two grades: mild to moderate regurgitation (regurgitation fraction ≤ 40%) and severe regurgitation (regurgitation fraction > 40%) [18]. Right ventricular function was obtained from MRI reports which were assessed by a cardiovascular radiologist (SS). Systolic function was expressed as right ventricular ejection fraction (RVEF) and graded as: normal (RVEF ≥ 55%), mildly impaired (RVEF ≥ 45% and <55%), moderately impaired (RVEF ≥ 35% and <45%), and severely impaired (RVEF < 35%).

BPA-RF and branch pulmonary distensibility were analyzed according to grading severity of right ventricular function.

The measurements were performed by a single observer. Every ninth case (n = 20) was selected to be measured by a second grader to estimate inter-observer agreements.

### 2.4. Statistical Analysis

Statistical analyses were performed using the STATA software version 16.0 (Stata Corp LLC, College Station, TX, USA). Continuous variables were tested for normality using Shapiro–Wilk test (Supplement S1). Due to departures from normality, descriptive data were presented as median (interquartile range (IQR), 25th–75th percentile). The normality data was presented as mean ± standard deviation (SD). Categorical variables were summarized as percentages. As a sensitivity analysis, we performed Huber robust regression, which is less dependent on assumptions of homoskedasticity and normally distributed residuals. We also analyzed children and adults separately in a subgroup analysis.

Differences were assessed using the Wilcoxon signed-rank test or paired *t* test. A logistic regression analysis was used to investigate the effect of pulmonary artery morphology on the BPA-RF, to assess the relationship between pulmonary insufficiency and BPA-RF and the correlation with branch pulmonary distensibility, and to identify whether branch pulmonary distensibility was associated with right ventricular dysfunction. A multilevel mixed-effects model adjusted to pulmonary insufficiency was used to test the difference of the effect of pulmonary insufficiency on the right and the left BPA-RF. A multilevel mixed-effects model was applied to determine the difference in the effect of BPR-RF on the right and left pulmonary artery distensibility. Linear regression analysis adjusted to pulmonary insufficiency was used to determine the association between BPA-RF and right ventricular function. A *p*-value of <0.05 was considered statistically significant. Inter-observer agreement was displayed as mean ± SD and 95% confidence interval (CI), using a Bland–Altman analysis.

The study was approved by the Institutional Human Research Ethics Committee.

## 3. Results

The demographic characteristics and hemodynamic data of 182 patients with rTOF are summarized in Table 1 and Table 2, respectively. Of the 182 patients (median age 17.1, IQR 12.5–21 years), there were 104 children (age < 18 years) (57.1%) and 78 adults (age ≥ 18 years) (42.8%). The median age for children was 13.1 years (IQR 10.3–15.9) and for adults was 21.6 years (IQR 19.3–27.7). The ventricular septal defect was closed in all patients. A total of 156 patients (85%) underwent transannular patch. The majority of patients had mildly impaired right ventricular function, accounting for 47% of cases. Severe pulmonary insufficiency was found in the main pulmonary artery and LPA with an incidence of 64% and 58%, respectively. RPA regurgitation fraction was mild to moderate, accounting for 64% of cases.

**Figure 4:**
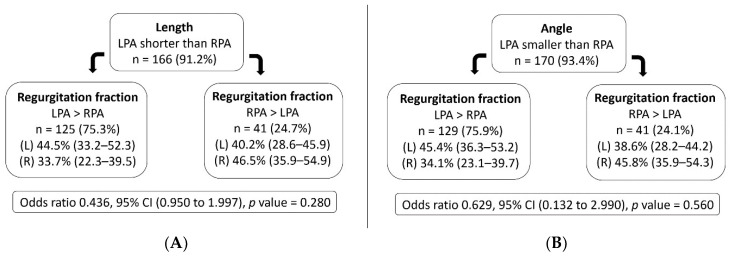
Effect of the length (**A**) and angle (**B**) of the left pulmonary artery on the branch pulmonary artery regurgitation, using linear regression analysis. Regurgitation fraction presented as median and interquartile range (25th–75th percentile).

The morphological characteristics and distensibility of the branch pulmonary arteries were significantly different. The LPA had a significant shorter length, a steeper angle, and a lesser distensibility compared with the RPA. The regurgitation fraction in the LPA was also significantly greater than the RPA, resulting in a significantly reduced net pulmonary blood volume to the left lung (Table 3). The effect of a shorter length and smaller angle of LPA on differential BPA-RF was assessed through a logistical regression analysis. Both morphologic features were not significant factors for the greater regurgitation fraction in the LPA. The odds ratio (95% CI), and *p*-values were as follows: length of 0.44 (0.95, 2.00), *p* = 0.28 and angle of 0.63 (0.13, 2.99), *p* = 0.56 (Figure 4A,B).

**Figure 5:**
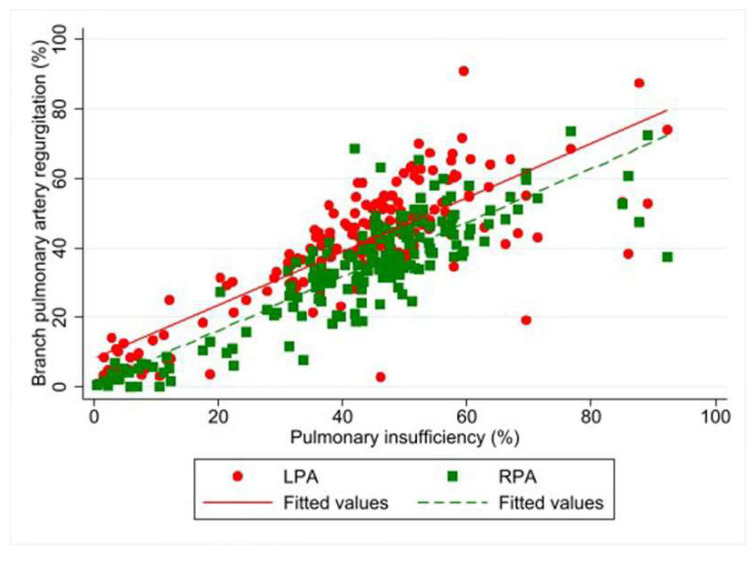
Correlation between pulmonary insufficiency and branch pulmonary artery regurgitation fraction, using linear regression analysis.

There was a positive correlation between pulmonary insufficiency (measured as regurgitation fraction) and BPA-RF. The median difference (95% CI), *p*-value was as follows: LPA of 0.78% (0.70, 0.86), *p* < 0.001 and RPA of 0.78% (0.71, 0.84), *p* < 0.001 (Figure 5). A significant greater regurgitation in the LPA than in the RPA was detected. The mean ± standard error was 40.8% ± 0.69 and 33.5% ± 0.69, respectively. The mean difference (95% CI), *p*-value was 7.32% (5.45, 9.20), *p* < 0.001.

**Figure 6:**
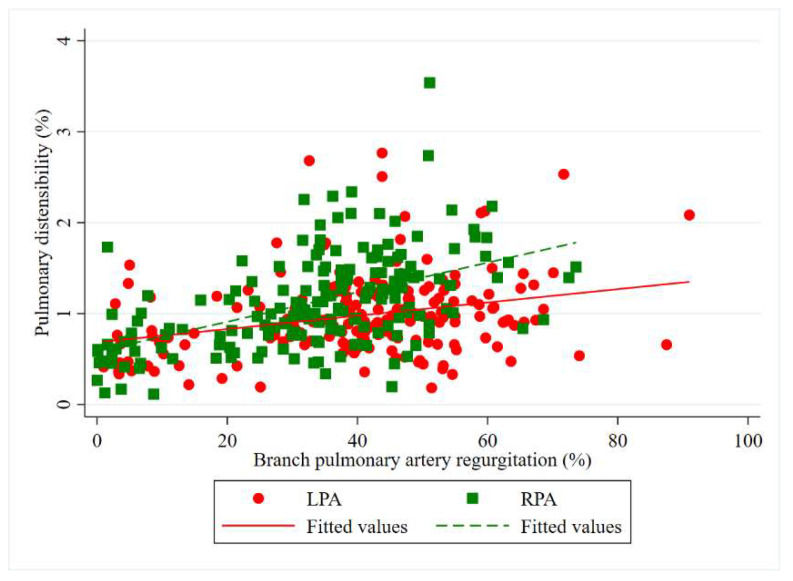
Correlation between branch pulmonary artery regurgitation fraction and branch pulmonary distensibility, using linear regression analysis.

Logistic regression analysis revealed a positive correlation between BPA-RF and branch pulmonary distensibility (Figure 6). Moreover, there was a statistically significant difference in distensibility between severe and non-severe regurgitation groups in both branch pulmonary arteries (Table 4). The median differences of distensibility in the LPA and RPA were 19.0% and 40.3%, respectively. The distensibility of the RPA was significantly greater than the LPA. The mean ± standard error was 112.9% ± 3.62 and 98.2% ± 3.62, respectively. The mean difference (95% CI), *p*-value was 14.68% (6.19, 23.19), *p* < 0.001.

Pulmonary insufficiency is a well-known risk factor for deterioration of right ventricular function. The adjusted BPA-RF and branch pulmonary distensibility were not significant factors for right ventricular dysfunction (Table 5).

Both Huber robust regression and subgroup analysis of children and adults demonstrated results similar to those seen with the overall data (n = 182). BPA-RF correlated with branch pulmonary distensibility significantly in both children and adults separately. RPA had a greater distensibility than the LPA (Supplementary Table S1). We also found that BPA-RF and branch pulmonary distensibility were not factors in determining right ventricular function in the overall data, in children alone, and in adults alone (Supplementary Table S2).

**Figure 7:**
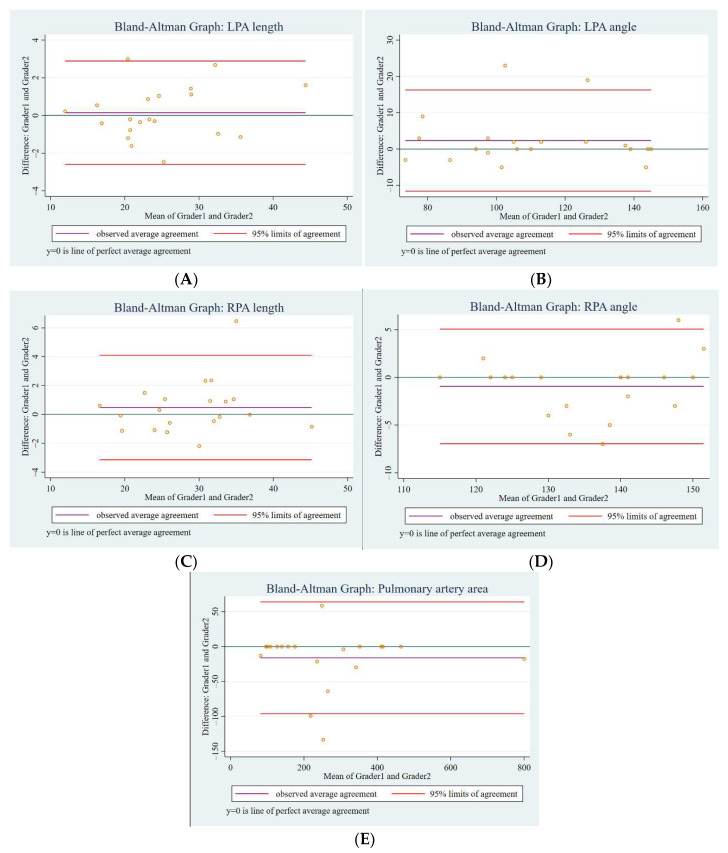
Bland–Altman plots of the inter-observer agreement in the measurements for LPA length (**A**), LPA angle (**B**), RPA length (**C**), RPA angle (**D**), and pulmonary area (**E**). The central violet line indicates the mean difference between the two graders. The upper and lower lines represent upper and lower margins of the 95% limits of agreement, respectively.

We randomly selected 20 patients for estimation of inter-observer agreement using Bland–Altman analysis. There were no significant differences between the two observers (mean difference (95% CI)) with details as followed: LPA length 0.15 mm (−2.60 to +2.89), LPA angle 2.35 degrees (−11.59 to +6.29), RPA length 0.48 mm (−3.14 to +4.11), RPA angle −0.95 degrees (−6.97 to +5.07), and pulmonary area −16.2 mm^2^ (−96.3 to +63.9) (Figure 7A–E).

## 4. Discussion

### 4.1. Pulmonary Artery Morphology, Distensibility, and Differential Branch Regurgitation Fraction

According to the current study, the LPA is significantly shorter in length and narrower in angle than the RPA. The regurgitation fraction in the LPA is far greater than in the RPA. The findings are comparable with a previous study (n = 22) which found that the regurgitation fraction in the LPA and RPA was 46 ± 18% and 39 ± 10%, respectively [9]. Nevertheless, our study found a greater regurgitation fraction in the RPA as compared with another study (n = 30) (35% vs. 29%) [11]. Voser et al. explained the observation of greater regurgitation in the LPA by the geometry of pulmonary artery bifurcation, and the smaller left lung volume with greater LPA impedance [11]. However, our data does not find a correlation between anatomical differences in the branch pulmonary arteries and differential branch regurgitation fraction. Therefore, the greater LPA impedance and non-laminar flow in the LPA are probably the possible explanations [8,19].

The normal distensibility in the RPA and LPA is 69 ± 21% in male and 53 ± 18% in female, and 39 ± 6% in male and 38 ± 7% in female, respectively [5]. Our study demonstrated that right and left pulmonary distensibilities in patients with rTOF are approximately 1.5 and 2.3 of normal values, respectively, while the previous study reported the greater branch distensibility in rTOF patients at about 1.3–1.4 times that of the control population (n = 16) [11]. The difference in the results could be related to the population number included in the studies (n = 182 vs. n = 30). The observation from our study could be explained by the pulmonary vascular response to increased intravascular volume from long-term pulmonary regurgitation.

Note that our study found greater distensibility in both pulmonary arteries compared with the previous study [11]. The difference is explained by the formula used. Our study used the formula described by Burman et al. where distensibility was derived from maximum area-minimum area × 100/minimum area [5], while the study of Voser et al. used the formula: maximum area–minimum area × 100/maximum area [11].

### 4.2. Relationship of Pulmonary Insufficiency to Branch Pulmonary Artery Regurgitation Fraction and Distensibility, and Their Association with Right Ventricular Function

Our study demonstrates a strong correlation between pulmonary insufficiency and BPA-RF. The LPA has a greater regurgitation fraction response than the RPA. We also observed a positive correlation between the BPA-RF and branch pulmonary distensibility. Moreover, the vascular distension capability is significantly greater in the RPA. The mechanism of this observation that LPA shows a greater regurgitation response to pulmonary insufficiency could be explained by the smaller left lung volume and a greater LPA impedance [7,8,9,11]. Although our findings suggest that branch pulmonary arteries in rTOF patients maintain vascular response to chronic increased blood volume by increasing wall distensibility, the lower LPA distension in response to regurgitation fraction should be explained. The possible mechanisms are chronic pulmonary insufficiency and a greater regurgitation fraction in the LPA, which causes a greater increased intravascular volume in the LPA than in the RPA. The more the arterial wall is stretched from its normal size, the stiffer the vessel wall is, and thus, decreases distensibility [20]. Therefore, patients with rTOF show a significantly lower net forward blood flow to the left lung compared with the right lung.

In addition, elastic fragmentation in the pulmonary artery commonly occurs in tetralogy of Fallot even before surgical correction [21]. Mechanical change during the loading of the pulmonary artery depends on both arterial elastin and collagen [22]. Differential branch pulmonary distensibility could be related to a difference in the amount of elastin and collagen. This hypothesis should be further investigated in future studies.

Only pulmonary insufficiency (regurgitation fraction) is a significant factor associated with right ventricular function. Both adjusted BPA-RF and branch pulmonary distensibility are not correlated with right ventricular dysfunction.

### 4.3. Study Limitation

There are limitations to this study. This was a single-center, retrospective study where the data may have been influenced by selection bias. Measurement of the length and angle of the pulmonary artery could be a source of error. However, this effect should have been minimized because we used multiple planes to determine the proper site to measure. Our study was limited to flow data and vascular distensibility, without determining the effect of pulmonary vascular resistance. Future studies are required to provide more details about the mechanisms of branch pulmonary regurgitation and distensibility.

## 5. Conclusions

This single-center, retrospective study of the BPA-RF was undertaken in 182 patients with rTOF, median age ranging from 12.5 years to 21.0 years. It has been shown that anatomical features of the branch pulmonary arteries are not a cause of the differential BPA-RF. There is a positive correlation between pulmonary insufficiency and BPA-RF, with a greater effect on the LPA. A strong relationship between BPA-RF and branch pulmonary distensibility suggests that pulmonary arteries in rTOF patients accommodate the increased intravascular volume by increasing distensibility. The net forward blood flow in the left lung is significantly less than in the right lung. Both BPA-RF and branch pulmonary distensibility are not determinants of right ventricular function.

## Figures and Tables

**Table 1:** Patient clinical characteristics (n = 182).

Characteristics	Values
Children (<18 years)	104 (57.1%)
Male sex	117 (64.3%)
Height (cm)	154.2 ± 17.0
Body weight (kg)	48.3 ± 17.1
Body surface area (m^2^)	1.73 ± 0.78
Age at MRI imaging (years)	17.1 (12.5–21.0)
Operative age (years) *	5 (4–7)
Surgical repair **	133 (73.1%)
Transannular patch	156 (85.7%)
Augmentation outflow	19 (10.4%)
Valve repair	5 (2.8%)
Unknown	2 (1.1%)

* n = 133. ** Repair with closure ventricular septal defect. Continuous variables presented as mean ± SD or median and interquartile range (25th–75th percentile) as appropriate, and categorical variables presented as n (%).

**Table 2:** Hemodynamic data.

Hemodynamic Data	Values
**Right ventricular ejection fraction (RVEF, %)**	**46.6 ± 8.4**
RVEF ≥ 55%	24 (13.2%)
RVEF ≥ 45% and <55%	86 (47.2%)
RVEF ≥ 35% and <45%	56 (30.8%)
RVEF < 35%	16 (8.8%)
**Pulmonary insufficiency (regurgitation fraction %)**	**45.3 (35.5–52.2)**
Mild to moderate degree (<40%)	65 (35.7%)
Severe degree (≥40%)	117 (64.3%)
**Left pulmonary regurgitation fraction (%)**	**43.1 (32.6–51.1)**
Mild to moderate degree (<40%)	75 (41.2%)
Severe degree (≥40%)	107 (58.8%)
**Right pulmonary regurgitation fraction (%)**	**35.2 (24.7–44.7)**
Mild to moderate degree (<40%)	84 (64.8%)
Severe degree (≥40%)	64 (35.2%)

Continuous variables presented as mean ± SD or median and interquartile range (25th–75th percentile) as appropriate, and categorical variables presented as n (%).

**Table 3:** Parameters of the left and right pulmonary arteries.

Variables	Left Pulmonary Artery	Right Pulmonary Artery	Coefficient (95% CI)	*p*-Value
Length (mm)	23.9 (20.7–27.5)	30.3 (26.0–34.9)	−6.4 ^a^ (−5.0, −7.8)	<0.001
Angle (degrees)	101.8 ± 18.3	141.0 ± 13.7	−39.2 ^b^ (−43.1, −6.0)	<0.001
Area-maximum (mm^2^)	238.1 (166.8–329.1)	247.8 (163.6–331.5)	-	0.661 *
Area-minimum (mm^2^)	119.4 (91.6–165.7)	110.7 (85.0–160.9)	-	0.024 *
Pulmonary distensibility (%)	92.9 (68.0–121.3)	105.5 (76.2–144.8)	-	0.001 *
Regurgitation fraction (%)	43.1 (32.6–51.5)	35.2 (24.7–44.7)	7.9 ^a^ (4.8, 11.0)	<0.001
Net pulmonary blood flow (mL)	21.0 (15.7–27.1)	31.3 (24.7–39.6)	−10.3 ^a^ (−7.7, 12.9)	<0.001

^a^ Median difference, ^b^ Mean difference; Data presented as mean ± SD or median and interquartile range (25th–75th percentile) as appropriate. Differences between left and right pulmonary arteries using paired *t*-test for normally distributed variables and Wilcoxon signed-rank test for non-normally distributed variables (*).

**Table 4:** Correlation between branch pulmonary artery regurgitation fraction and branch pulmonary distensibility.

Variables	Regurgitation Fraction (%)	Grading Branch Pulmonary Regurgitation Fraction (%)		Pulmonary Distensibility (%) Coefficients (95% CI)	*p*-Value
	Total	Mild to Moderate	Severe		
Left pulmonary artery	n = 182 (100%)	n = 75 (41.2%)	n = 107 (58.8%)	0.73 ^a^ (0.37, 1.09)	<0.001
	43.1 (32.6–51.1)	29.7 (10.2–37.1)	50.1 (44.5–57.6)	19.02 ^b^ (6.08, 31.95)	0.004
Right pulmonary artery	n = 182 (100%)	n = 118 (64.8%)	n = 64 (35.2%)	1.63 ^a^ (1.22, 2.03)	<0.001
	35.2 (24.7–44.7)	30.3 (13.1–34.9)	47.2 (43.9–53.2)	40.33 ^b^ (23.47, 57.20)	<0.001

^a^ Correlation between branch pulmonary artery regurgitation fraction and pulmonary distensibility by using linear regression analysis (n = 182). ^b^ Median difference of left pulmonary artery distensibility comparing between mild-to-moderate and severe branch pulmonary regurgitation fraction by using median regression analysis. Data presented as median and interquartile range (25th–75th percentile) and categorical variables presented as n (%).

**Table 5:** Factors associated with right ventricular function.

Factors (%)	Total	Grading Right Ventricular Ejection Fraction (RVEF, %)				*p*-Value
		≥55%	≥45% and <55%	≥35% and <45%	<35%	
	n = 182 (100%)	n = 24 (13.2%)	n = 86 (47.2%)	n = 56 (30.8%)	n = 16 (8.8%)	
RVEF (mean ± SD)	46.6 ± 8.4	59.1 ± 2.9	50.1 ± 3.1	40.4 ± 2.9	30.6 ± 3.6	<0.001
Pulm-insuff (mean ± SD)	45.3 (35.5–52.2)	37.6 (10.9–48.0)	43.1 (31.7–50.6)	47.1 (39.6–56.4)	53.2 (46.6–67.6)	0.042
LPA distensibility median (IQR)	92.9 (68.0–121.3)	88.2 (70.8–129.2)	97.4 (71.3–123.6)	82.8 (64.6–108.6)	99.8 (83.4–125.6)	0.383
RPA distensibility median (IQR)	105.5 (76.2–144.8)	88.4 (57.7–120.6)	103.5 (76.4–144.9)	115.4 (76.3–144.2)	129.0 (95.4–157.4)	0.368
LPA-RF * (mean ± SE)	40.8 ± 0.68	40.5 ± 2.14	41.6 ± 1.11	41.1 ± 1.39	35.9 ± 2.64	0.268
RPA-RF * (mean ± SE)	33.5 ± 0.68	32.1 ± 1.71	32.9 ± 0.89	34.3 ± 1.11	35.9 ± 2.11	0.434

* Data derived from linear regression analysis adjusted pulmonary insufficiency. Data presented as mean ± SD or median and interquartile range, IQR (25th–75th percentile), and categorical variables presented as n (%) as appropriate. LPA = left pulmonary artery, LPA-RF = left pulmonary artery regurgitation fraction, RPA = right pulmonary artery, RPA-RF = right pulmonary artery regurgitation fraction, Pulm-insuff = pulmonary insufficiency, SE = standard error.

## Data Availability

Data sharing is not applicable to this article.

## Supplementary Materials

The following are available online at https://www.mdpi.com/article/10.3390/tomography7030036/s1. Supplement S1. Test the normality of data. Supplementary Table S1. Correlation between branch pulmonary artery regurgitation fraction and branch pulmonary distensibility. Supplement Table S2. Factors associated with right ventricular function.

## Abbreviations

BPA-RF	Branch pulmonary artery regurgitation fraction
LPA	Left pulmonary artery
MRI	Magnetic resonance imaging
PA	Pulmonary artery
PI	Pulmonary insufficiency
RF	Regurgitation fraction
rTOF	Repaired tetralogy of Fallot
RPA	Right pulmonary artery
RVF	Right ventricular function
RVEF	Right ventricular ejection fraction
