# Supplementary material for: Branch Pulmonary Artery Regurgitation in Repaired Tetralogy of Fallot: Correlation with Pulmonary Artery Morphology, Distensibility, and Right Ventricular Function
Source: Tomography. 2021 Sep 1;7(3):412–23. doi: 10.3390/tomography7030036 (PMC8482212; doi:10.3390/tomography7030036)
Supplement: Supplementary file 1 [file tomography-07-00036-s001.zip › tomography-1314007-supplementary.pdf]

Supplementary tables

Supplement S1. Test the normality of data

**Factors**	***p*-value**
Age	<0.001
Left pulmonary artery distensibility	<0.001
Right pulmonary artery distensibility	<0.001
Left pulmonary artery regurgitation fraction	<0.001
Right pulmonary artery regurgitation fraction	<0.001
Right ventricular ejection fraction	0.415*
Pulmonary insufficiency	<0.001
Left pulmonary artery- length	0.027
Right pulmonary artery- length	<0.001
Left pulmonary artery- angle	0.579*
Right pulmonary artery- angle	0.158*
Net pulmonary blood flow- left pulmonary artery	<0.001
Net pulmonary blood flow- right pulmonary artery	<0.001

* Data is normal distribution

Supplementary Table S1. Correlation between branch pulmonary artery regurgitation fraction and branch pulmonary distensibility.

Variables		Regurgitation fraction (%)	Grading branch pulmonary regurgitation fraction (%)		Pulmonary distensibility (%)	*p*-value
		Total	Mild to moderate	Severe	Coefficients (95% CI)	
**Overall** (n = 182)	Left pulmonary artery	n = 182 (100 %)	n = 75 (41.2 %)	n = 107 (58.8 %)	0.73[a] (0.37, 1.09)	< 0.001
		43.1 (32.6 - 51.1)	29.7 (10.2 - 37.1)	50.1 (44.5 - 57.6)	19.02[b] (6.08, 31.95)	0.004
	Right pulmonary artery	n = 182 (100 %)	n = 118 (64.8 %)	n = 64 (35.2 %)	1.63[a] (1.22, 2.03)	< 0.001
		35.2 (24.7 - 44.7)	30.3 (13.1 - 34.9)	47.2 (43.9 - 53.2)	40.33[b] (23.47, 57.20)	< 0.001
**Children** (<18 years) (n = 104)	Left pulmonary artery	n = 104 (57.1 %)	n = 41 (39.4 %)	n = 63 (60.6 %)	0.54[a] (0.06, 1.04)	0.028
		43.1 (31.9 - 52.1)	28.6 (12.6 - 36.1)	49.4 (44.2 - 55.0)	17.85[b] (0.25, 35.45)	0.047
	Right pulmonary artery	n = 104 (57.1 %)	n = 64 (61.5 %)	n = 40 (38.5 %)	1.73[a] (1.19, 2.29)	< 0.001
		35.5 (24.4 - 45.7)	29.5 (11.4 - 34.3)	46.8 (44.7 - 51.0)	42.30[b] (20.85, 63.75)	< 0.001
**Adults** (≥18 years) (n = 78)	Left pulmonary artery	n = 78 (42.9 %)	n = 34 (43.6 %)	n = 44 (56.4 %)	0.94[a] (0.39, 1.49)	0.001
		43.0 (34.6 - 51.4)	30.8 (9.6 - 38.6)	51.0 (45.3 - 58.9)	19.69[b] (-0.68, 40.07)	0.058
	Right pulmonary artery	n = 78 (42.9 %)	n = 54 (69.2 %)	n = 24 (30.8 %)	1.47[a] (0.87, 2.09)	< 0.001
		35.2 (25.3 - 43.2)	31.0 (18.3 - 36.0)	48.0 (43.6 - 58.0)	33.21[b] (-4.39, 70.81)	0.083

[a] Correlation between branch pulmonary artery regurgitation fraction and pulmonary distensibility by using linear regression analysis (n = 182). [b] Median difference of left pulmonary artery distensibility comparing between mild-to-moderate and severe branch pulmonary regurgitation fraction by using median regression analysis. Data presented as median and interquartile range (25th - 75th percentile) and categorical variables presented as n (%).

Supplement Table S2. Factors associated with right ventricular function.

	Factors (%)	Total	Grading right ventricular ejection fraction (RVEF, %)				*p*-value
			≥ 55 %	≥ 45 % and < 55 %	≥ 35 % and < 45 %	< 35 %	
		**n = 182 (100 %)**	**n = 24 (13.2 %)**	**n = 86 (47.2 %)**	**n = 56 (30.8 %)**	**n = 16 (8.8 %)**	
**Overall** (n = 182)	RVEF (mean ± SD)	46.6 ± 8.4	59.1 ± 2.9	50.1 ± 3.1	40.4 ± 2.9	30.6 ± 3.6	< 0.001
	Pulm-insuff median (IQR)	45.3 (35.5 - 52.2)	37.6 (10.9 - 48.0)	43.1 (31.7 - 50.6)	47.1 (39.6 -56.4)	53.2 (46.6 - 67.6)	0.042
	LPA distensibility median (IQR)	92.9 (68.0 - 121.3)	88.2 (70.8 - 129.2)	97.4 (71.3 - 123.6)	82.8 (64.6 - 108.6)	99.8 (83.4 - 125.6)	0.383
	RPA distensibility median (IQR)	105.5 (76.2 - 144.8)	88.4 (57.7 - 120.6)	103.5 (76.4 - 144.9)	115.4 (76.3 - 144.2)	129.0 (95.4 - 157.4)	0.368
	LPA-RF* (mean ± SE)	40.8 ± 0.68	40.5 ± 2.14	41.6 ± 1.11	41.1 ± 1.39	35.9 ± 2.64	0.268
	RPA-RF * (mean ± SE)	33.5 ± 0.68	32.1 ± 1.71	32.9 ± 0.89	34.3 ± 1.11	35.9 ± 2.11	0.434
**Children** (<18 years) (n = 104)	**Factors (%)**	**n = 104 (100 %)**	**n = 13 (12.5 %)**	**n =53 (51.0 %)**	**n = 27 (26.0 %)**	**n = 11 (10.5 %)**	***p*-value**
	RVEF (mean ± SD)	46.2 ± 8.5	58.2 ± 1.5	49.7 ± 3.0	40.1 ± 2.9	29.8 ± 3.9	< 0.001
	Pulm-insuff median (IQR)	45.3 (34.3 – 52.1)	44.1 (11.2 - 48.0)	42.2 (31.4 - 48.4)	49.0 (41.9 - 57.4)	63.8 (46.6 - 69.5)	0.022
	LPA distensibility median (IQR)	99.5 (73.3 - 126.1)	93.7 (76.2 – 132.6)	100.7 (73.2 - 125.8)	91.3 (65.8 - 116.8)	112.4 (87.1 – 126.4)	0.701
	RPA distensibility median (IQR)	106.1 (82.2 - 144.4)	100.2 (60.5 - 132.8)	103.9 (79.9 - 143.9)	114.9 (93.3 - 143.5)	131.1 (97.3 - 163.1)	0.580
	LPA-RF* (mean ± SE)	40.6 ± 0.83	40.9 ± 2.73	41.2 ± 1.35	42.2 ± 1.91	33.6 ± 3.1	0.108
	RPA-RF * (mean ± SE)	33.6 ± 0.83	32.2 ± 2.02	33.2 ± 1.00	34.9 ± 1.41	34.2 ± 2.29	0.703
**Adults** (≥18 years) (n = 78)	**Factors (%)**	**n = 78 (100 %)**	**n = 11 (14.1 %)**	**n = 33 (42.3 %)**	**n = 29 (37.2 %)**	**n = 5 (6.4 %)**	***p*-value**
	RVEF (mean ± SD)	47.1 ± 8.3	60.1 ± 3.8	50.5 ± 3.3	40.7 ± 2.8	32.3 ± 2.3	< 0.001
	Pulm-insuff median (IQR)	45.6 (35.7 - 52.2)	36.7 (6.9 - 47.8)	46.1 (36.1 - 54.0)	45.9 (37.6 - 52.9)	49.2 (44.7 - 52.2)	0.580
	LPA distensibility median (IQR)	88.3 (64.0 - 109.3)	87.8 (55.8 - 121.3)	93.1 (71.3 - 113.3)	76.5 (56.7 – 97.0)	92.7 (64.9 - 97.9)	0.589

RPA distensibility median (IQR)	101.4 (69.3 - 144.8)	82.5 (56.8 - 116.8)	103.1 (70.5 - 146.7)	115.9 (69.7 - 144.8)	126.8 (93.6 - 151.8)	0.619
LPA-RF* (mean ± SE)	41.0 ± 1.1	40.1 ± 3.47	42.3 ± 1.94	40.0 ± 2.08	41.0 ± 5.01	0.871
RPA-RF * (mean ± SE)	33.3 ± 1.1	32.0 ± 2.97	32.7 ± 1.66	33.5 ± 1.78	38.6 ± 4.29	0.607

* Data derived from linear regression analysis adjusted pulmonary insufficiency. Data presented as mean ± SD or median and interquartile range, IQR (25th - 75th percentile), and categorical variables presented as n (%) as appropriate. LPA = left pulmonary artery, LPA-RF = left pulmonary artery regurgitation fraction, RPA = right pulmonary artery, RPA-RF = right pulmonary artery regurgitation fraction, Pulm-insuff = pulmonary insufficiency, SE = standard error.
